# Supplementary material for: Learning curve and surgical outcome of robotic assisted colorectal surgery with ERAS program
Source: Sci Rep. 2022 Nov 29;12:20566. doi: 10.1038/s41598-022-24665-w (PMC9709162; doi:10.1038/s41598-022-24665-w)
Supplement: Supplementary file 2 — Supplementary Information 2. [file 41598_2022_24665_MOESM2_ESM.zip › RAL Raw data-Ñ[▒K/097/OP N_ñw╝╨░O▒Kñσ.pdf]

W92 / W92

臺中榮民總醫院
手術室護理記錄

[redacted]

基本資料

- 室別： 開刀房-17室，手術科別： CRS，手術類別： 常規，日期： 2021/07/09
- 進入時間： 1500 ； 麻醉開始時間： 1515 ； Time Out時間： 1615 ； 劃刀時間： 1616 ； 手術結束時間： 2005 送出時間： 2025 ；
- 麻醉方式： GE

  診斷：Malignant tumor of A-colon

  手術名稱：Robot Assisted Colectomy(R)

  手術局部用藥：NIL

  術前預防性抗生素：
  - 抗生素名稱一： Veterin 1GM IVD/由麻醉護理人員給予
  - 時間： 1605
  - 記錄者： 林家羽

  ，追加預防性抗生素：
- 手術醫師： 蔣鋒帆，陳映菁，陳映晴
- 麻醉醫師： VS 齊光彪
-
  - 刷手護士A ： 許雅芳，時間： 1500，刷手護士B ： 羅媗鈴，時間： 1500，接替刷手一： 賴雯惠，時間： 1600，接替刷手二： 盧陵怡，時間： 1800
  - 流動護士A ： 林家羽，時間： 1500，接替流動一： 陳家瑋，時間： 2000
- 更多接替及流動護士： 接替刷手三： 蘇敬涵，時間： 1900，接替刷手四： 王明盈，時間： 2000

皮膚狀況

A-V Shunt： 無

術前： 完整

記錄者： 上次簽章: 林家羽

術後： 完整

記錄者： 上次簽章: 陳家瑋

手術準備

手術臥式 單一手術臥式

-
  - 手術臥式： 仰臥
  - 使用附件： 頭圈，墊肩，其它： Silicon pad*1、海綿枕、頭架
  - 皮膚準備： 10%Aq Betadine，Hibiscrub，2% Chlorhexidine
  - 電燒： 使用電燒: 電極片位置： 其它： 電燒墊，記錄者： 上次簽章: 林家羽

止血帶T： 無

植入物

- 植入物名稱/號碼/數量/部位：GIA/ 80MM-3.8MM/2/Colon

，記錄者： 上次簽章: 林家羽

標本送檢

- 細菌培養 無
- 細胞學 無
- 冰凍切片 無
- 病理標本

  組織名稱一：A-colon
- 截肢 無

標本交付 標本： 1 袋，標本： 0 盒，標本： 0 瓶，細胞學： 0 管，Culture Tube： 0 管，被交付醫師： R 陳映晴，

- 記錄者： 上次簽章: 陳家瑋

- 時間： 2005

傷口術後數料

傷口數料：

- 請於圖上標示部位：

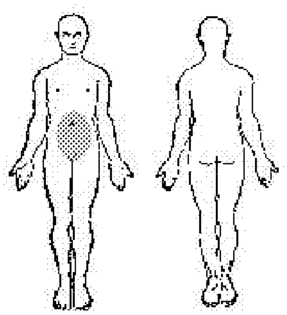

troca

- 傷口數料： 軟性紗布

導管

導管	天數	導管資訊	插入日期	插入時間	更換日期	拔除日期	拔除時間	拔除原因	預計拔除/更換	預計拔除/更換日期
Foley(1)	1	號碼:16 Fr.; 部位:膀胱; 註記:10cc;	2021/07/09	1600						
Epidural PCA(1)	1	部位:硬脊外;	2021/07/09	1505						
Double Lumen CVC(1)	1	號碼:8Fr ; 部位:; 象限:RU; 註記:15CM;	2021/07/09	1525						
A-line (1)	1	號碼:20G ; 部位:左手;	2021/07/09	1530						

用物計數

- 手術中出血量： minimal c.c.
- 手術中尿量： 200 c.c.
-
  - 其它： 無

用物計數： 多切口同時縫合

- 1.器械： 正確，2.縫針： 正確，3.刀片： 正確，4.紗布： 正確，5.腹部墊： 未使用，6.紗球： 未使用，7.PEANUTS ： 未使用，8.紗條： 未使用，9.絲帶： 未使用，10.針頭： 未使用，11.Vessel loop： 未使用，12.Boots： 未使用，13.Jelco： 未使用，14.棉條： 未使用，
  - 第一次：
    - 刷手護士 ： 蘇敬涵
    - 流動護士： 林家羽
    - 時間： 1935

  ,
  - 第二次：
    - 刷手護士 ： 蘇敬涵
    - 流動護士： 林家羽
    - 時間： 1945

  ,
  - 第三次：
    - 刷手護士 ： 蘇敬涵
    - 流動護士： 林家羽
    - 時間： 1950

備註

備註： 17-2 Davinci 10mm30度

- 記錄者： 上次簽章: 林家羽
- 時間： 1500

填寫護理人員 林家羽

正式摘要

W92 / W92

doctorRank:B
